# MYCN and MAX alterations in Wilms tumor and identification of novel N-MYC interaction partners as biomarker candidates

**DOI:** 10.1186/s12935-021-02259-2

**Published:** 2021-10-24

**Authors:** Ovidio Jiménez Martín, Andreas Schlosser, Rhoikos Furtwängler, Jenny Wegert, Manfred Gessler

**Affiliations:** 1 Theodor-Boveri-Institute/Biocenter, Developmental Biochemistry, Wuerzburg University, 97074 Wuerzburg, Germany; 2 Rudolf Virchow Center, Center for Integrative and Translational Bioimaging, Wuerzburg University, 97078 Wuerzburg, Germany; 3 Department of Pediatric Oncology Und Hematology, Saarland University Hospital, 66421 Homburg, Saar Germany; 4 Comprehensive Cancer Center Mainfranken, Wuerzburg University, 97078 Wuerzburg, Germany

**Keywords:** Wilms tumor, *MYCN*, *MAX*, Interactome, Mutation screening

## Abstract

**Background:**

Wilms tumor (WT) is the most common renal tumor in childhood. Among others, *MYCN* copy number gain and *MYCN* P44L and *MAX* R60Q mutations have been identified in WT. *MYCN* encodes a transcription factor that requires dimerization with MAX to activate transcription of numerous target genes. *MYCN* gain has been associated with adverse prognosis in different childhood tumors including WT. The *MYCN* P44L and *MAX* R60Q mutations, located in either the transactivating or basic helix-loop-helix domain, respectively, are predicted to be damaging by different pathogenicity prediction tools, but the functional consequences remain to be characterized.

**Methods:**

We screened a large cohort of unselected WTs for *MYCN* and *MAX* alterations. Wild-type and mutant protein function were characterized biochemically, and we analyzed the N-MYC protein interactome by mass spectrometric analysis of N-MYC containing protein complexes.

**Results:**

Mutation screening revealed mutation frequencies of 3% for *MYCN* P44L and 0.9% for *MAX* R60Q that are associated with a higher risk of relapse. Biochemical characterization identified a reduced transcriptional activation potential for *MAX* R60Q, while the *MYCN* P44L mutation did not change activation potential or protein stability. The protein interactome of N-MYC-P44L was likewise not altered as shown by mass spectrometric analyses of purified N-MYC complexes. Nevertheless, we could identify a number of novel N-MYC partner proteins, e.g. *PEG10*, *YEATS2*, *FOXK1*, *CBLL1* and *MCRS1,* whose expression is correlated with *MYCN* in WT samples and several of these are known for their own oncogenic potential.

**Conclusions:**

The strongly elevated risk of relapse associated with mutant *MYCN* and *MAX* or elevated *MYCN* expression corroborates their role in WT oncogenesis. Together with the newly identified co-expressed interactors they expand the range of potential biomarkers for WT stratification and targeting, especially for high-risk WT.

**Supplementary Information:**

The online version contains supplementary material available at 10.1186/s12935-021-02259-2.

## Background

Wilms tumor (WT) is the most common pediatric renal tumor, with an incidence of approximately 1 in 10.000 children. In Europe, patients are treated according to the International Society of Pediatric Oncology (SIOP) protocol, consisting of preoperative chemotherapy, followed by surgery, and risk-adjusted postoperative chemo- and radiotherapy [1]. Although the overall survival rate is at 92%, blastemal histology in chemotherapy-treated WTs is associated with adverse prognosis and reduced relapse-free survival. Genetic characterization of this histological subgroup is therefore of clinical relevance, in order to find biomarkers for risk-stratification or potential therapeutic leads.

In previous exome sequencing studies of high-risk blastemal WTs, we and others identified several potential oncogenic driver mutations of genes involved in miRNA biogenesis and kidney development [2–5]. Among these, alterations in the *MYCN* and *MAX* genes were detected: predominantly *MYCN* copy number gain (mostly low copy amplification), but also the somatic variants *MYCN* P44L and *MAX* R60Q. It has been reported that *MYCN* gain correlates with anaplasia and reduced relapse-free and overall survival in WT [6], but it is also associated with poor outcome in other pediatric cancers such as medulloblastoma, neuroblastoma and rhabdomyosarcoma [7]. *MYCN* P44L and *MAX* R60Q mutations are classified as pathogenic by different functional prediction tools. They have since been detected at low frequencies in several other tumor types like neuroblastoma, glioma, and some carcinomas [8]. In total *MYCN* alterations affected up to 18.5% of Wilms tumors treated with preoperative chemotherapy, suggesting an important oncogenic function of *MYCN* [2].

The N-MYC protein, encoded by the *MYCN* gene, is a member of the MYC family, a small group of basic helix-loop-helix leucine zipper (bHLH-LZ) transcription factors (TFs) that heterodimerize with MAX. These heterodimers bind to E-box motifs (CACGTG) and regulate a multitude of cellular functions, like cell proliferation, cell cycle control, differentiation and apoptosis [7]. The biochemical effects of both mutations, *MYCN* P44L and *MAX* R60Q, remained unclear, however. P44L is located within the N-MYC transactivation domain (TAD), preceding the so-called Myc-box I that carries a phosphodegron (T58/S62) regulating N-MYC stability and activity. The R60Q mutation affects the MAX bHLH domain, required for protein dimerization and DNA-binding (Additional file 1: Figure S1).

To better characterize the role of *MYCN* and *MAX* mutations in WT, we first screened a larger cohort of cases to evaluate possible clinic-pathological correlations. We then assayed the biochemical functions and protein interactions of the mutant proteins with a focus on N-MYC.

## Materials and methods

### Patient materials

Wilms tumor and control tissues with associated clinical data were obtained from the German SIOP93-01/GPOH and SIOP2001/GPOH studies. Informed consent had been obtained for tumor banking and future research use according to German regulations (Ethikkommission der Ärztekammer des Saarlandes, 136/01). DNA and RNA were isolated as described before [9].

### Verification of SNVs

Allele-specific PCR (ASP) for the *MYCN* P44L and *MAX* R60Q mutation was performed using primers designed with WebSNAPER (https://pga.mgh.harvard.edu/cgi-bin/snap3/websnaper3.cgi). Samples with known mutations were used as controls. Potential variants were verified by Sanger sequencing. Expression of mutant alleles was checked by RT-PCR of DNaseI treated RNA, followed by Sanger sequencing. To screen for further MAX variants, the entire coding region was amplified from tumor cDNA with primers in the 5’- and 3’-UTR. Primers are listed in Additional file 6: Table S6.

### Expression vectors

Cloning of expression vectors was done using primers listed in Additional file 6: Table S7 to amplify coding regions from plasmids or cDNA from HEK293 cells or tumor material. The pGL3-6XEBOX-prom luciferase reporter vector was generated by inserting six E-box sites [10] upstream of the SV40 promoter of the pGL3-Promoter vector (Promega). All constructs were verified by Sanger sequencing.

### Cell culture and transfections

HEK293 and U2OS cells were cultured in DMEM / 10% FCS, 50 U Penicillin and 50 µg/ml Streptomycin, and transfected using polyethyleneimine. For stable transfection, the doxycycline-inducible vectors pSB-ET-iE-HA-MYCN (wildtype or P44L mutant) and pSB-ET-iE-FLAG-MAX (wildtype or R60Q mutant) were introduced via the Sleeping Beauty transposase system with puromycin selection (Additional file 5: Figure S5). Stably transfected clones were titrated with doxycycline, to ensure minimal and equal expression of N-MYC or MAX among biological replicates, verified by Western blot analysis. Final concentrations are listed in Additional file 6: Table S8. Doxycycline-induction of transiently transfected cells (500 ng/ml) was started 6–12 h after transfection, and for all transfected cells induction was carried for 48 h.

### Luciferase assay

10^5^ HEK293 or U2OS cells were seeded on 24-well-plates and transiently transfected in triplicates, using pGL3-6XEBOX-prom as the luciferase reporter. Luciferase activity was measured as described previously [11], using a Berthold Tristar multimode reader.

### Protein stability assay

5 × 10^5^ stably transfected HEK293 cells were plated on 6-well-plates. After 48 h of induction, the culture medium was exchanged and 100 µM cycloheximide (Roth) and/or 20 µM MG-132 inhibitor (Biomol) were added. Following incubation, cells were washed with PBS and whole cell lysates were assayed by Western blot.

### MTT assay

2000 control or stably transfected cells were seeded in triplicates in a 96-well plate. After induction with or without doxycycline, 5 mg/ml MTT (3-(4,5-dimethylthiazol-2-yl)-2,5-diphenyltetrazolium bromide, Sigma-Aldrich) was added to the cells for 2 h. Cells were then lysed for 20 min with 150 μl DMSO, and accumulation of formazan was quantified at 590 nm in a Berthold Tristar multimode reader.

### Quantitative real-time RT–PCR (qRT–PCR)

qRT-PCR was performed as described before [9], using primers listed in Additional file 6: Table S9. Briefly, 1 µg RNA was treated with DNAseI and reverse transcribed using random hexamer primers (Thermo Fischer). PCR was performed with 1/25 of a cDNA reaction with SybrGreen quantification and melting curve validation of products. The housekeeping gene HPRT was used to normalize expression levels. For the analysis of gene expression in WT samples, three reference cDNAs were used on each PCR plate to ensure the comparability of values between plates.

### Co-immunoprecipitation (co-IP)

Native N-MYC complexes were isolated following a published protocol [12]. 10–12 million HEK293 cells (200 million for MS analysis), were induced for 48 h and harvested in ice cold PBS supplemented with 50 µg/ml PMSF (Applichem), 1 µg/ml aprotinin, leupeptin and pepstatin (Sigma-Aldrich), and Phosphatase Inhibitor Cocktails II and III (1:10,000 dilution, Sigma-Aldrich). Cell pellets were resuspended in lysis buffer (20 mM HEPES pH 7.9, 180 mM NaCl, 1.5 mM MgCl_2_, 10% glycerol and 0.2% Nonidet P40) containing fresh inhibitors, homogenized 15 times using 27G needles (or by douncing in case of large cell pellets), followed by sonicating 4 × 10 s with 45 s pausing (20% output). Benzonase (100 U/ml; Novagen) was added and the sample was incubated for 40 min at 4 °C. Insoluble material was pelleted by centrifugation (18,000 rpm, 30 min, 4 °C). The soluble protein fraction obtained from 10–12 million HEK293 cells was split and was used for IP with 20 µl of HA-coupled magnetic beads (Pierce Thermo Fisher Scientific) or FLAG-coupled agarose beads (Sigma), with additional 15 U benzonase per reaction. For MS analysis, the soluble protein fraction obtained from 200 million HEK293 cells was mixed with 80 μl HA-coupled magnetic beads and 200 units benzonase per reaction. Beads were incubated at 4 °C with rotation, 3 h for HA-beads and overnight for FLAG-beads. HA-beads were washed 3 × at 4 °C in lysis buffer containing 0.1% Triton X-100 and then twice in buffer without Triton X-100. FLAG-beads were washed 6 times at 4 °C in co-IP lysis buffer. Beads were resuspended in 30 µl 1 × SDS loading buffer (0.1 M Tris pH 6.8, 4% SDS, 0.25% bromophenol blue, 25% glycerol and 10% 2-mercaptoethanol) or 100 µl 1 × NuPAGE® LDS Sample Buffer (Thermo Fisher Scientific) in case of MS samples, and incubated at 95 °C for 5–10 min. For the analysis of post-translational modifications of N-MYC, cells were lysed in RIPA buffer (50 mM Tris pH 8.0, 1% Nonidet P40, 0.5% sodium deoxycholate, 0.1% SDS, 150 mM NaCl and 1 mM EDTA) to reduce unspecific contaminants.

### Western blot

For Western blot analysis, lysates from equivalent numbers of cells were directly lysed in 1 × Laemmli sample buffer (100 µl per 10 cm plate). 10% of the cell lysates, or one sixth of the resuspended Co-IP beads were run on 12% polyacrylamide gels and blotted on nitrocellulose membranes. Blots were blocked with 5% milk powder, sequentially incubated with primary and horseradish‐peroxidase‐conjugated secondary antibodies (Additional file 6: Table S10) in PBS or TBST and developed with X‐ray‐film (Super RX‐N, Fuji Medical X‐Ray Film). Vinculin served as a loading control in all cases.

### Sample preparation for quantitative mass spectrometry

Precipitation of wild-type and mutant HA-N-MYC was performed overnight at -20 °C with a fourfold volume of acetone. Pellets were washed three times with acetone at -20 °C. Precipitated proteins were dissolved in NuPAGE® LDS sample buffer (Life Technologies), reduced with 50 mM DTT at 70 °C for 10 min and alkylated with 120 mM Iodoacetamide at room temperature for 20 min. Separation was performed on NuPAGE® Novex® 4–12% Bis–Tris gels (Life Technologies) according to manufacturer’s instructions. Gels were washed three times for 5 min with water and stained for 45 min with Simply Blue™ Safe Stain (Life Technologies). After washing with water for 2 h, gel lanes were cut into 15 slices. For PTM analysis, only the area corresponding to the N-MYC protein size was used. Excised gel bands were destained with 30% acetonitrile in 0.1 M NH_4_HCO_3_ (pH 8), shrunk with 100% acetonitrile, and dried in a vacuum concentrator. Digests were performed with 0.1 µg trypsin (or chymotrypsin, for PTM analysis) per gel band overnight at 37 °C in 0.1 M NH_4_HCO_3_ (pH 8). After removing the supernatant, peptides were extracted from the gel slices with 5% formic acid, and extracted peptides were pooled with the supernatant.

NanoLC-MS/MS analyses were performed on an Orbitrap Fusion (Thermo Scientific) equipped with a PicoView Ion Source (New Objective), coupled to an EASY-nLC 1000 (Thermo Scientific). Peptides were loaded on capillary columns (PicoFrit, 30 cm × 150 µm ID, New Objective) self- packed with ReproSil-Pur 120 C18-AQ, 1.9 µm (Dr. Maisch) and separated with a 30 min linear gradient from 3 to 30% acetonitrile and 0.1% formic acid and a flow rate of 500 nl/min.

Both MS and MS/MS scans were acquired in the Orbitrap analyzer with a resolution of 60,000 for MS scans and 15,000 for MS/MS scans. HCD fragmentation with 35% normalized collision energy was applied. A Top Speed data-dependent MS/MS method with a fixed cycle time of 3 s was used. Dynamic exclusion was applied with a repeat count of 1 and an exclusion duration of 30 s; singly charged precursors were excluded from selection. Minimum signal threshold for precursor selection was set to 50,000. Predictive AGC was used with AGC a target value of 2e5 for MS scans and 5e4 for MS/MS scans. EASY-IC was used for internal calibration.

### MS data analysis

Raw MS data files were analyzed with MaxQuant version 1.6.2.2 [13]. Database search was performed with Andromeda, integrated in MaxQuant, against the UniProt Human database. Additionally, a database containing common contaminants was used. The search was performed with tryptic cleavage specificity with 3 allowed miscleavages. Protein identification was under control of the false-discovery rate (1% FDR on protein and peptide level). In addition to MaxQuant default settings, the search was performed against the following variable modifications: Protein N-terminal acetylation, Gln to pyro-Glu formation (N-term. Gln) and oxidation (Met). Carbamidomethyl (Cys) was set as fixed modification. For protein quantitation, the LFQ intensities were used [14]. Proteins with less than two identified razor/unique peptides were dismissed. Further data analysis was performed using R scripts developed in-house. Missing LFQ intensities in the control samples were imputed with values close to the baseline. Data imputation was performed with values from a standard normal distribution with a mean of the 5% quantile of the combined log10- transformed LFQ intensities and a standard deviation of 0.1. For the identification of significantly co-immunoprecipitated proteins, boxplot outliers were identified in intensity bins of at least 300 proteins. Log2 transformed protein ratios of co-IP versus control (Log2FC) with values outside a 1.5x (potential) or 3x (extreme) interquartile range (IQR), respectively, were considered as significantly co-immunoprecipitated.

Data analysis for phosphorylation site identification of wild type and P44L mutant HA-N-MYC was performed with PEKAS Studio X (Bioinformatics Solution Inc., Canada). Database searching was performed against a custom database containing the protein sequence of HA-N-MYC with the following parameters: parent mass tolerance: 8 ppm, fragment mass tolerance: 0.02 Da, enzyme: chymotrypsin, variable modifications: oxidation (M), pyro-glutamate (N-term. Q), Protein N-term acetylation, phosphorylation (STY). Results were filtered to 1% PSM-FDR by target-decoy approach, and MS/MS spectra of phosphopeptides were validated manually.

### Gene ontology analysis

Gene ontology analysis of the extreme outliers obtained from the MS of wild-type and mutant HA-N-MYC was performed using a PANTHER Overrepresentation Test [15], allowing only to enrich for cellular location. The Homo sapiens reference list was used, as well as the False Discovery rate (FDR) correction method to account for multiple hypothesis testing.

### Statistics

Statistical analyses were performed using the two-tailed Fisher’s exact test or the Mann-Whitney U test for continuous variables. Kaplan Meier plot were generated using the log rank test (SPSS Version 13.0).

## Results

### *MYCN* and *MAX* mutation screening

To determine the incidence and possible clinical or pathological implications of the *MYCN* P44L and *MAX* R60Q mutations we screened a cohort of unselected WTs using allele-specific PCR. Patient data included sex, age, presence of familial WT or predisposition syndromes as well as metastasis, relapse, and survival (with a follow-up of > 2 years).

**Fig. 1:**
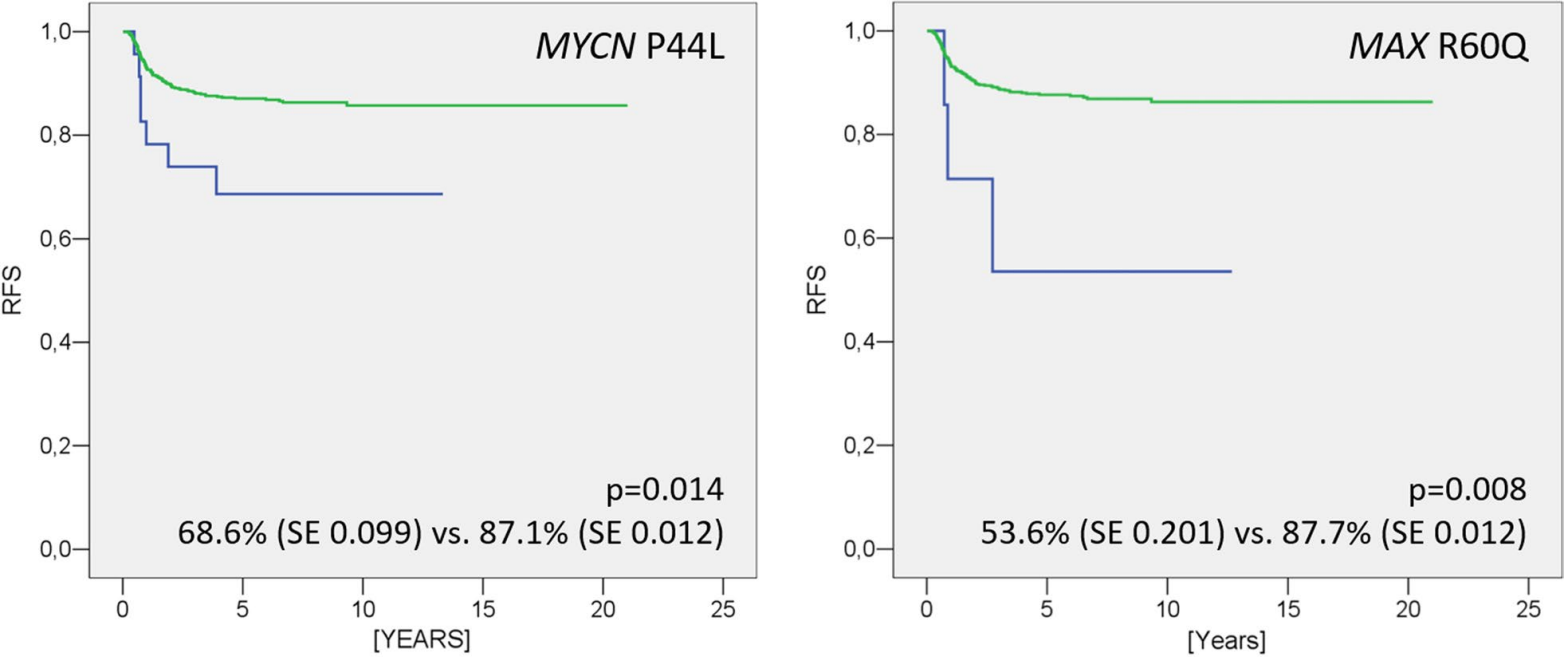
Relapse Free Survival Estimates for *MYCN* and *MAX* mutant tumors. Relapse Free Survival Estimates were calculated for *MYCN* P44L (left) and *MAX* R60Q (right) according to the method of Kaplan–Meier and compared using the log rank test (SPSS Version 13.0). p <  = 0.05 were considered significant

*MYCN* P44L was identified in 24 of 810 WT patients (3%) (Table 1). All cases carried heterozygous somatic mutations with an allele frequency between 10 and 50%, except for a single homozygous case (Additional file 6: Table S1). The mutation was detected in most histological subtypes, most frequently in the blastemal subtype (Table 1). No association was found with sex, age at diagnosis, or histological subtype. There was a highly significant correlation between *MYCN* P44L status and relapse, especially local relapse, however: 10.9% of cases harboring the mutation suffered a local relapse, compared to 2.4% of the non-mutated cases. Kaplan–Meier curves document this striking difference in relapse free survival (Fig. 1).

**Table 1:** *MYCN* P44L and *MAX* R60Q mutation frequency in clinical and histological subgroups

	Clinical data				Histology*									Total cases*
					Low-risk	Intermediate-risk						High-risk		
	Metasatic relapse	Local relapse	Relapse (any)	Death	Compl. necrotic	Epithelial	Stromal	Mixed	Regressive	Focal anaplasia	Blastemal, primary surgery	Blastemal	Diffuse Anaplasia	
*MYCN* P44L	4/85	6/55 ^#^	7/108 ^§^	3/44	0/31	3/81	1/80	6/231	9/267	0/15	1/25	4/61	0/34	24/810
(%)	4.7%	10.9%	6.5%	6.8%	–	3.7%	1.3%	2.6%	3.4%	–	4.0%	6.6%	–	3.0%
*MAX* R60Q	2/77	2/51	3/99 ^§^	1/40	0/30	0/80	0/77	2/225	4/255	0/15	0/24	1/57	0/34	7/782
(%)	2.6%	3.9%	3.0%	2.5%	–	–	–	0.9%	1.6%	–	–	1.8%	–	0.9%

^*^825 (*MYCN* P44L) and 797 (*MAX* R60Q) tumors from 810 and 782 cases, respectively. For 15 bilateral cases both histotypes were assessed

# p = 0.005; §p < 0.05 (two-tailed Fisher's exact test)

The incidence of *MAX* R60Q was lower with 7 of 782 cases (0.9%) [Table 1] harboring a somatic mutation. Allele frequencies only reached 5–30% in sequence chromatograms (Additional file 6: Table S1). In four tumors with multiple biopsies, percentages ranged from 0 to 30% mutant allele per each case, while complete allele loss (LOH) for markers on chromosome 11p or 16q assured high tumor cell content even for MAX wild-type specimens (data not shown). This indicates that the *MAX* R60Q mutation must be present in just a fraction of tumor cells, occurring as a late event. The mutation was observed in tumors with regressive, mixed, and blastemal histology (Table 1). *MAX* R60Q had a higher incidence in relapsing (3 vs. 0.5%) cases with again significant differences in survival curves (Fig. 1).

No other recurrent *MYCN* or *MAX* mutation has been described in WT to date. In hereditary pheochromocytoma additional *MAX* mutations were found, however [16]. We therefore sequenced most of the coding sequence (aa 22–160) in a set of 101 WT cDNAs. Only two silent N125N variants (c.375C > T) were found. Thus, R60Q remains the only functional *MAX* alteration observed in WT.

The tumor cohorts had been screened in parallel for *DROSHA* E1147K mutations that are found in high-risk blastemal WT ([2] and R. Vardapour, pers. comm.). There was a co-occurrence of *MYCN* P44L and *DROSHA* E1147K, but it did not reach statistical significance (p = 0.0869) (Additional file 6: Table S2).

### Transactivation and dimerization of MAX-R60Q and N-MYC-P44L

**Fig. 2:**
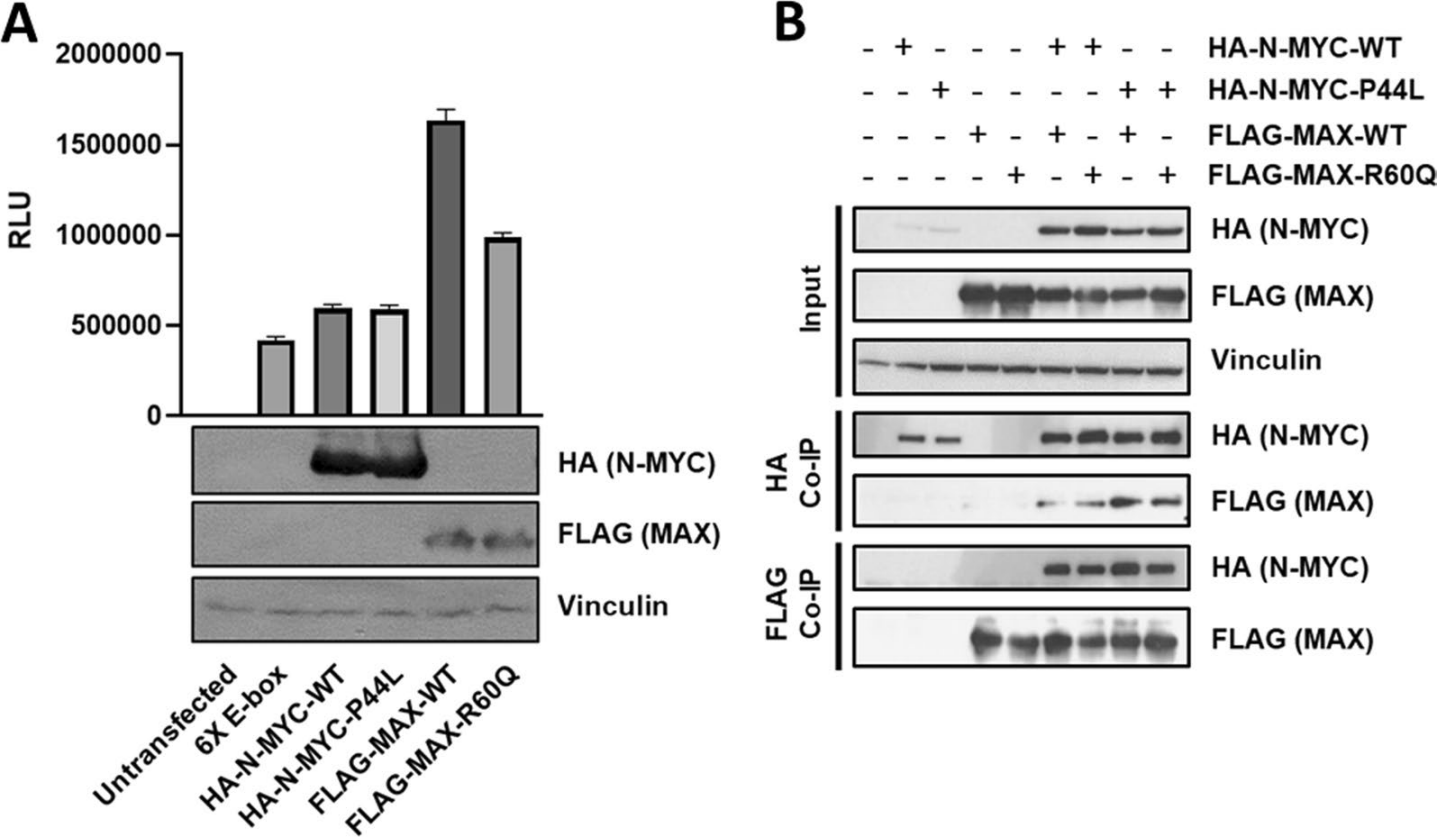
Transactivation and dimerization of MAX-R60Q and N-MYC-P44L. **A** Luciferase activity (RLU) in HEK293 cells, transiently transfected with a reporter construct containing 6 E-boxes (6X E-box), and the wild-type or mutant HA-N-MYC or FLAG-MAX expression vectors or empty vector control. In the lower panel, corresponding Western blots confirm protein expression with Vinculin as control. **B** Immunoblots of co-IP experiments. Wild-type and mutant HA-N-MYC and FLAG-MAX were expressed by transient transfection in HEK293 cells. Lysates from input control and the corresponding immunoprecipitates were tested by Western blot

For functional characterization of *MYCN* P44L and *MAX* R60Q, we measured the activation of a luciferase reporter vector containing 6 canonical E-box motifs. Transient transfection in HEK293 cells led to a comparable, 40% increase in luciferase activity by wild-type and mutant N-MYC (Fig. 2A). MAX led to a fourfold increase in luciferase activity, but this increase was blunted by the R60Q mutation. This indicates a reduced transactivation potential, in line with the location of the amino acid exchange within the helix-loop-helix domain needed for dimerization. Comparable results were obtained in U2OS cells, indicating that this is a general phenomenon (data not shown).

The impact of the mutations on dimer formation was tested by co-immunoprecipitation (co-IP) of N-MYC/MAX heterodimers (Fig. 2B). We did not detect differences in binding of mutant compared to wild-type dimers under these conditions, which may be too subtle to be detectable in this assay, but consistent with the partly retained transactivation capacity of the mutants.

Interestingly, even low-level induction of N-MYC led to a compensatory reduction in C-MYC protein and cell proliferation ceased after 2 days of induction of either wild-type and P44L mutant N-MYC (Additional file 2: Figure S2). The third paralog, MYCL is not expressed according to RNA-seq data [17]. This reduced growth may be reconciled with known pro-apoptotic function of MYC protein overexpression [18]. Interestingly, this was not seen with MAX or MAX-R60Q overexpression, which was well tolerated (data not shown).

### N-MYC interactome

To evaluate a possible effect of *MYCN* P44L on protein interaction, we performed mass spectrometry (MS) on HA-tagged wild-type and P44L mutant N-MYC complexes, purified from stably transfected HEK293 cells. The quality of the IP elutions was assessed by Western blot and silver staining (Additional file 3: Figure S3), before proceeding with label-free quantification MS analysis. Two biological replicates were performed for wild-type and P44L mutant, respectively.

**Fig. 3:**
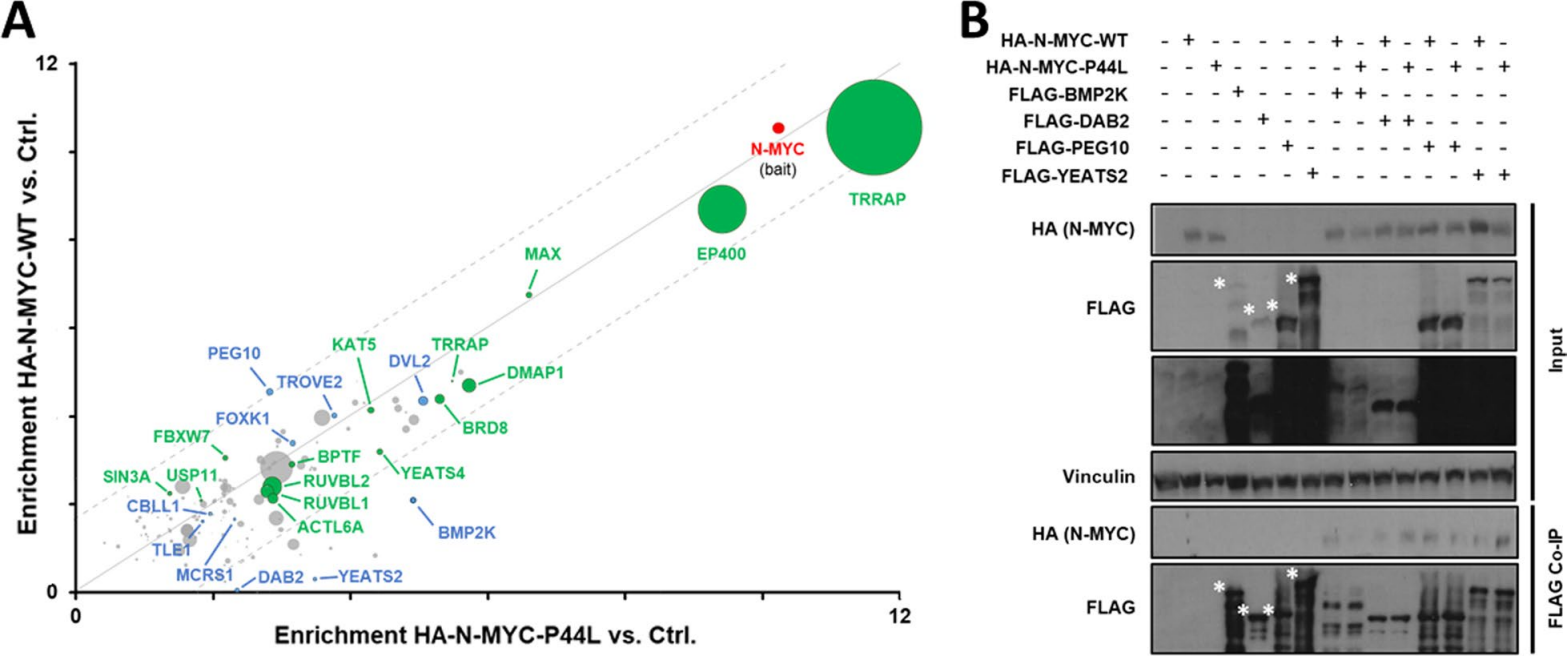
Comparison of the wild-type and N-MYC-P44L interactome. **A** Summary of proteins enriched in wild-type and P44L mutant N-MYC complexes. The x axis displays the enrichment (log2 fold-change) of proteins in wild-type HA-N-MYC-expressing cells compared to control cells, while the y axis displays the enrichment in HA-N-MYC-P44L expressing cells, both calculated from the mean of biological duplicates. Previously characterized N-MYC interactors are depicted in green, novel interactors that were tested via immunoprecipitation in blue. The size of dots correlates with the number of identified razor and unique peptides of the corresponding protein. Dots located close to the solid diagonal line represent proteins equally enriched in both complexes, while dots beyond the dotted lines were more strongly enriched either in the wild-type or the mutant complexes. **B** Immunoblots of HEK293 cells transiently transfected with expression vectors for HA-N-MYC and candidates for which binding to N-MYC is potentially affected by the P44L mutation. White asterisks represent the specific bands of N-MYC interactor candidates in the input and FLAG co-IP. Vinculin input served as a loading control

Only proteins that were significantly co-immunoprecipitated in at least one replicate were included for further analysis, resulting in 140 interactors (Fig. 3A; Table 2) (see Additional file 6: Table S3 for full listing). These included several known N-MYC interactors, like its dimerization partner MAX, members of chromatin-remodeling complexes required for MYC-mediated transcriptional regulation, e.g. EP400 or TRRAP [19], FBXW7, involved in N-MYC proteasomal degradation [20], and the ubiquitin-specific protease USP11, required for recruitment of BRCA1 and enhancement of transcriptional activation [21]. Gene Ontology (GO) analysis of the extreme outliers (values lying more than 3 times the interquartile range below the first quartile or above the third quartile) showed a significant enrichment of nuclear and chromatin-related proteins, as well as chromatin remodeling complex constituents (Additional file 6: Table S4).

**Table 2:**
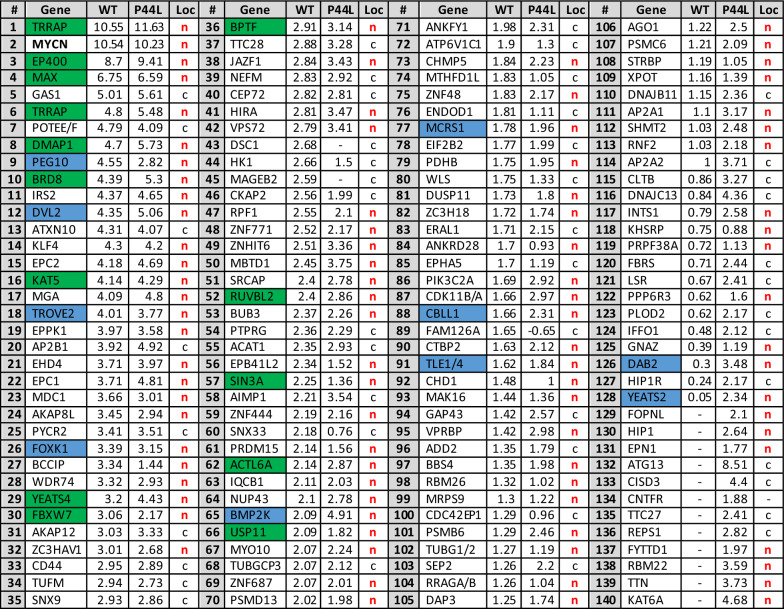
Top 140 enriched protein in wild-type and P44L mutant N-MYC complexes

Characterized N-MYC interactors are depicted in green, novel interactors that were tested via immunoprecipitation in blue. Enrichment (log2FC) and cellular location according to the GeneCards database (n = nucleus and c = cytosol) are indicated

There was only a small number of 21 proteins that appeared to be differentially bound by the two N-MYC variants. Candidates with the highest enrichment in either wild-type or mutant N-MYC complexes and known nuclear location (based on the GeneCards database) were chosen for validation via co-IP: BMP2K, DAB2, PEG10 and YEATS2 (Fig. 3A). None of them had previously been reported to bind N-MYC. Their interaction with N-MYC as novel partners could be confirmed, but we could not verify a differential binding due to the mutation (Fig. 3B).

### Validation of new N-MYC interactors

**Fig. 4:**
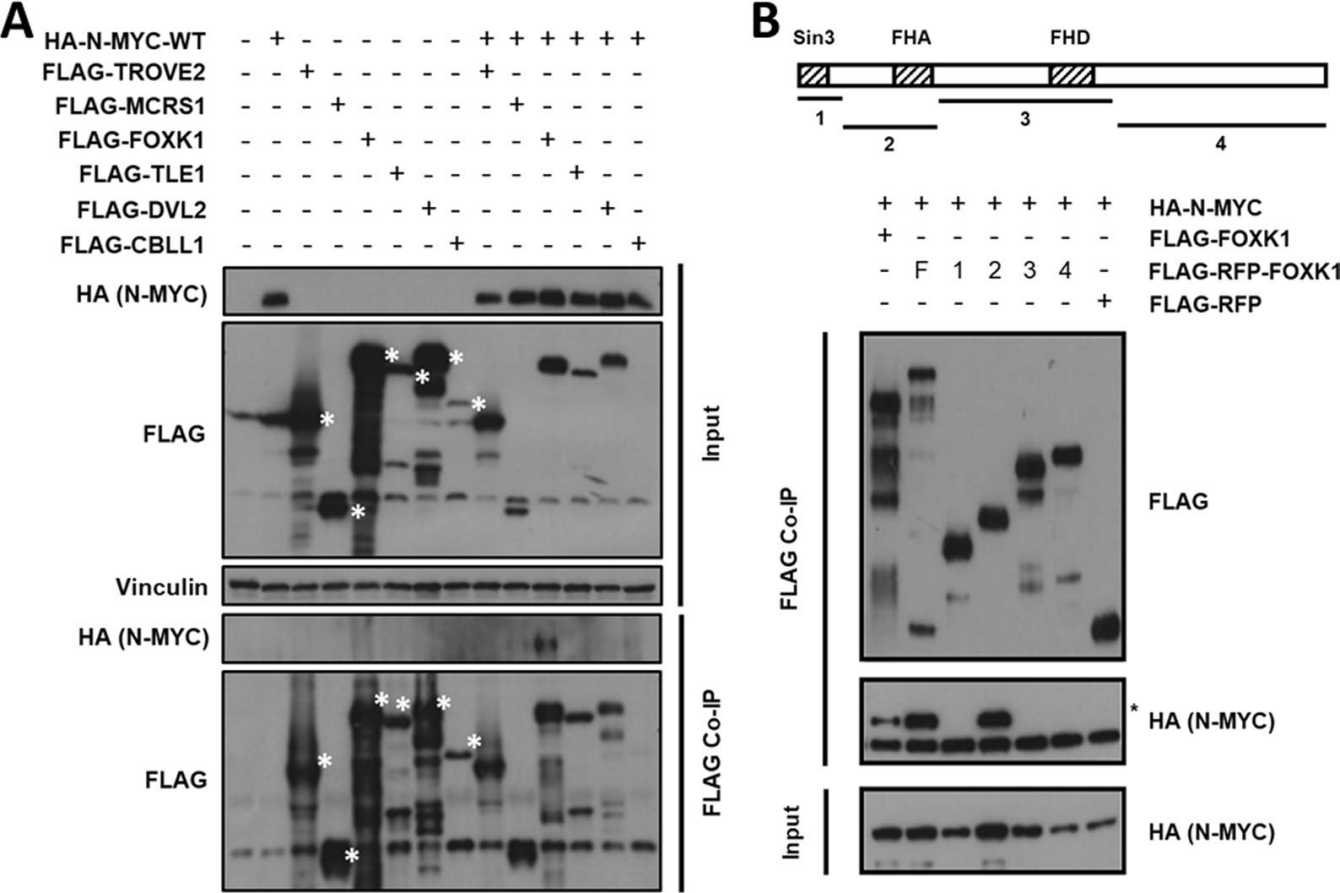
Characterization of novel N-MYC interactors. **A** Immunoblots of HEK293 cells transiently transfected with expression vectors for HA-N-MYC and potential novel N-MYC interaction partners. White asterisks identify the specific bands for N-MYC interactors. **B** Scheme of FOXK1 deletion mutants and co-IP of HA-N-MYC with flag-tagged FOXK1 deletion mutants in HEK293 cells. The asterisk in FLAG-IP blot marks the band corresponding to HA-N-MYC protein. F: full-length FOXK1, 1–4: FOXK1 deletion mutant vectors

Among our collection of 140 N-MYC interactors, we identified 45 nuclear proteins that had not been reported to bind N-MYC before. We selected six candidates for validation by co-IP: DVL2, TROVE2, and the proto-oncogene proteins FOXK1, TLE1, CBLL1 and MCRS1. The interaction of N-MYC with FOXK1, MCRS1 and CBLL1 could be confirmed with variable strength (Fig. 4A). This suggests that their association with N-MYC is more dynamic or limited to certain complexes. Nevertheless, our analysis widens the already broad spectrum of N-MYC interactors.

We further characterized the stronger FOXK1/N-MYC interaction since the related FOXR2 has been shown before to bind C-MYC and to promote cell proliferation and oncogenic transformation [22]. Deletion analysis of RFP-FOXK1 fusions revealed a strong interaction of N-MYC with the forkhead-associated domain (FHA) of FOXK1, a phosphopeptide recognition domain that could provide readouts of N-MYC phosphorylation (Fig. 4B).

### Concerted expression of N-MYC and its interactors

**Fig. 5:**
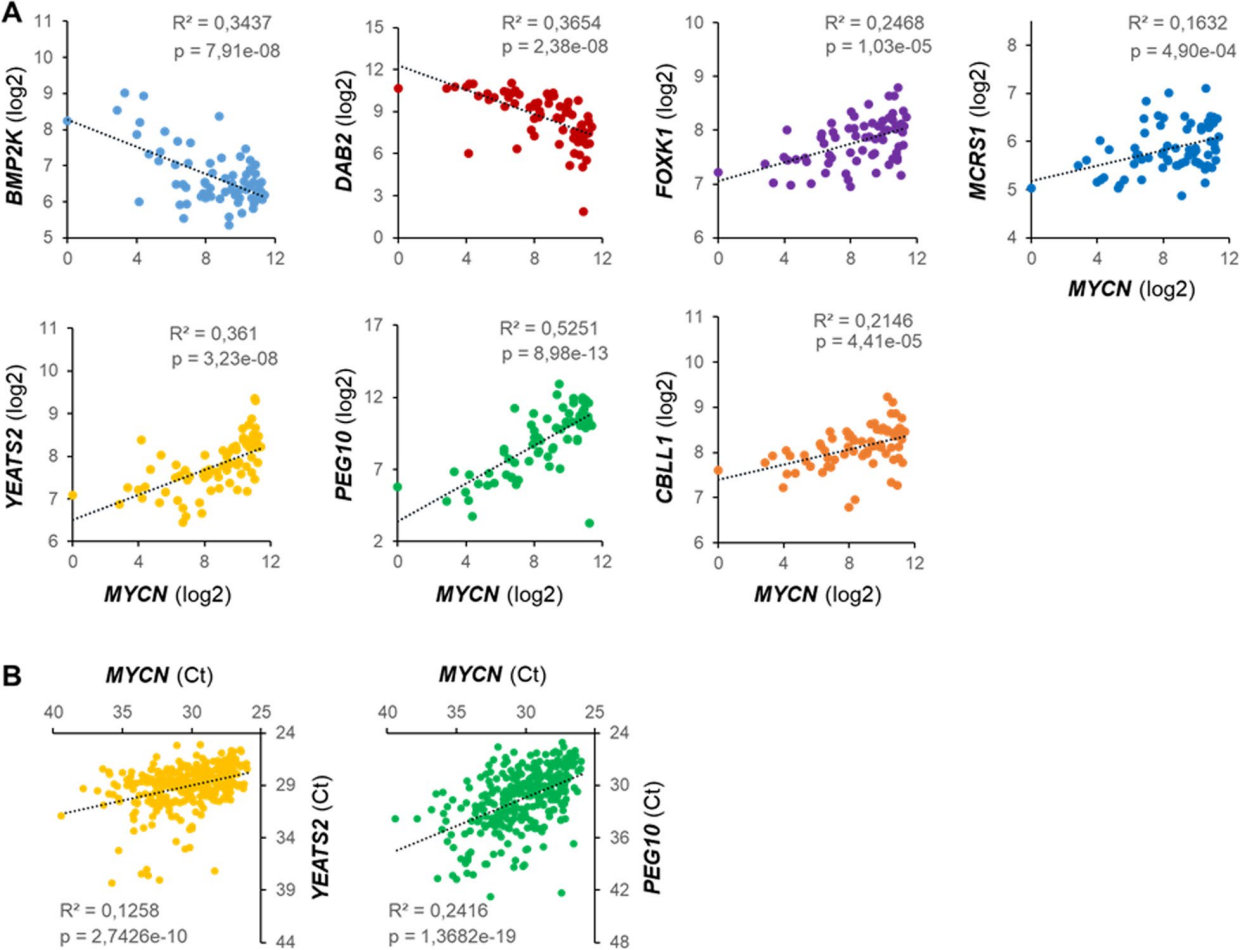
Expression of *MYCN* and interactors in Wilms tumor. **A** Scatter plots showing mRNA expression (log2-fold) of *MYCN* (x axes) and interaction partners (y axes) in WT (data from Wegert et al., 2015 [2]), visualized by R2: Genomics Analysis and Visualization Platform (http://r2.amc.nl). For each plot, the coefficient of determination (R^2^) and p-value (p) are listed. **B** Scatter plots with cycle threshold (Ct) values, representing the correlation between *MYCN* (x-axis) and *PEG10*/*YEATS2* expression (y-axes) in 299 WTs. Coefficients of determination (R^2^) and p-values (p) are indicated

These newly found N-MYC interacting proteins may be directly relevant to Wilms tumors as suggested by their concerted expression. Five of seven proven candidates, *PEG10*, *YEATS2*, *FOXK1*, *CBLL1* and *MCRS1* showed a clear positive correlation of mRNA levels with *MYCN* in Wilms tumors undergoing the SIOP protocol [2] (Fig. 5A). Similar results were obtained for datasets of 224 favorable histology Wilms tumors [23] and 649 neuroblastoma cases [24] (data not shown).

Extended expression analysis in our own independent cohort of 299 Wilms tumors by qRT-PCR confirmed the correlated expression of *PEG10* and *YEATS2* with *MYCN* (Fig. 5B). Especially *MYCN* and *PEG10* showed very similar patterns of expression in different subtypes of Wilms tumors, predominantly the high-risk blastemal type, where *YEATS2 *was also significantly overexpressed (Additional file 6: Table S5). This data set also confirmed the prior association of higher *MYCN* levels with fatal outcome (p < 0.001), but this was not seen for its interacting partners.

### N-MYC-P44L phosphorylation status and half-life

**Fig. 6:**
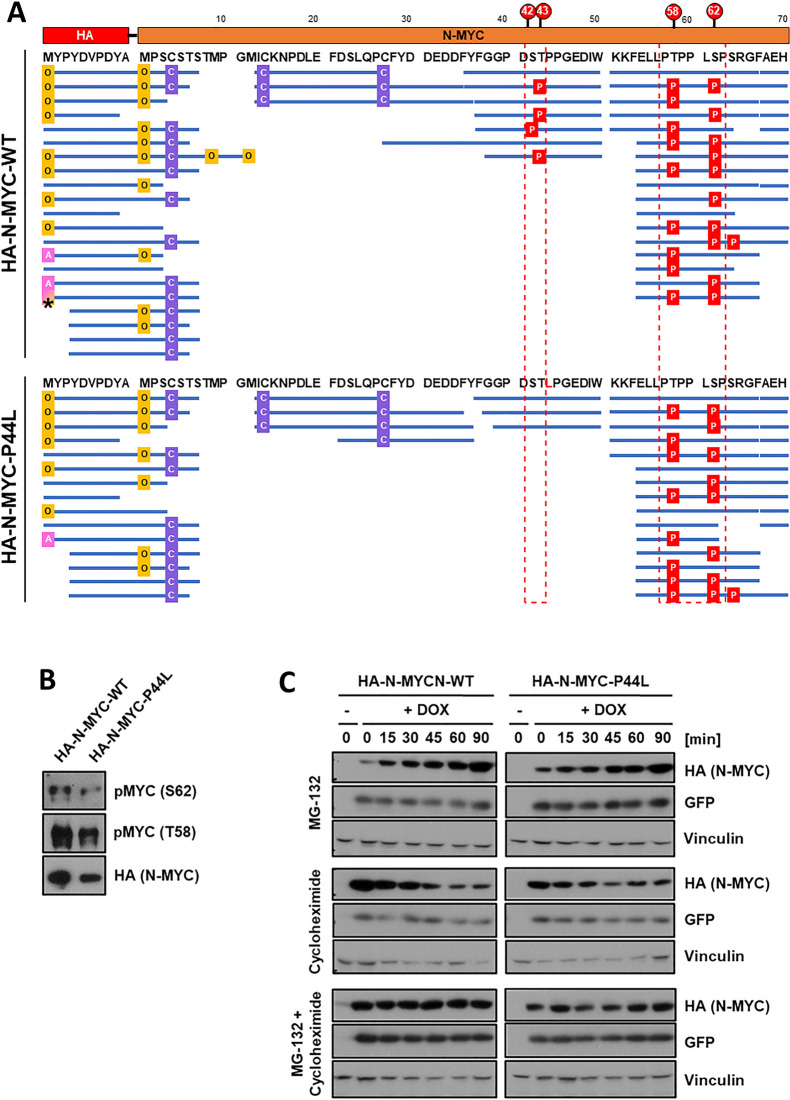
N-MYC-P44L phosphorylation status and stability. **A** Graphical summary of the results of MS peptide analyses. The amino-termini of wild-type and P44L mutant HA-N-MYC are depicted with the corresponding amino acid residues and positions in the top. Blue lines represent peptides identified by MS after digestion with chymotrypsin. Identified protein modifications included phosphorylation (P, in red), oxidation of methionine (O, in yellow), protein N-terminal acetylation (A, in pink) and carbamidomethylation (C, in violet). Dashed boxes indicate the phosphorylated residues identified by MS, corresponding to positions S42, T43, T58 and S62. **B** Western blot analysis showing phosphorylation status of T58 and S62 in HA-N-MYC. **C** Immunoblots from protein stability assays of wild-type and mutant HA-N-MYC in stably transfected HEK293 cells. Inhibitor treatment with MG-132 and / or cycloheximide was performed for 0–90 min as indicated. *GFP* expression is coupled to *MYCN* via an IRES sequence (see Fig. S5)

N-MYC is subject to strong post-translational regulation and its stability can be modulated through phosphorylation at positions T58 and S62 that are part of the N-MYC phosphodegron. To evaluate the influence of the P44L mutation on T58/S62 or the adjacent candidate phosphorylation sites S42 and T43, we analyzed N-MYC peptides for post-translational modifications (PTM) by MS (Figs. 6A and Additional file 4: Figure S4A). Wild-type and mutant HA-N-MYC derived peptides presented a similar ratio of phosphorylation at residues T58 and S62. This could be validated by Western blot analysis using antibodies against T58- and S62-phosphorylated MYC (Fig. 6B). At position S42/T43 we detected a novel, frequent phosphorylation in wild-type N-MYC. However, the N-MYC-P44L derived peptides showed a lack of phosphorylation at this position, likely due to the loss of the recognition site of a presumed proline-directed kinase (NetPhos prediction of CDK5 or CKII [25]) (Fig. 6A). Extracted ion chromatograms obtained from these peptides confirm the complete lack of phosphorylation at S42/T43 in N-MYC-P44L (Additional file 4: Figure S4B-C).

To assess if this lack of phosphorylation could influence N-MYC degradation, we assessed its protein stability. Inhibition of de novo protein synthesis and proteasome degradation by cycloheximide and MG-132, respectively, yielded comparable stability for wild-type and mutant N-MYC (Fig. 6C). Thus, the P44L mutation apparently neither influences the T58/S62 phosphodegron of N-MYC nor its stability, but prevents phosphorylation at S42/T43, whose implications are yet to be revealed.

## Discussion

MYC proteins regulate a range of cellular processes and their dysregulation has a large impact on the development of cancer: different types of *MYC* and *MYCN* alterations have been identified in a large variety of cancers and they are often correlated with poor prognosis and reduced survival [7]. This makes MYC proteins not only significant targets for novel therapeutic approaches in cancer, but relevant biomarkers for early risk-stratification of patients.

## *MYCN*/*MAX* alterations as risk factors

In WT it was mostly copy number gain of *MYCN* [6] and elevated expression [26] that were linked to reduced survival. Exome sequencing recently revealed point mutations of *MYCN* (P44L) and its heterodimer partner *MAX* (R60Q) in WT [2, 6, 27]. We have now performed the largest screening for *MYCN* P44L and *MAX* R60Q using 810 WT patients. *MYCN* P44L was identified in 3% of cases, similar to previous studies. The *MAX* R60Q mutation had a frequency of 0.9%, slightly lower than reported before (1.7%) [27] and it was the only relevant alteration to be found in the *MAX* coding region, unlike in pheochromocytoma [16]. *MAX* R60Q mutations appear to be late clonal events suggested by their variable presence in multiply sampled tumors. Both, *MYCN* and *MAX* mutations were significantly associated with relapse, which may make them valuable additions to biomarkers for the prediction of clinical course.

Diffuse anaplasia is the strongest clinical predictor of poor outcome in WT. Interestingly, neither *MYCN* nor *MAX* mutations were found in 34 diffuse and 15 focal anaplastic tumors. In the American COG cohort *MYCN* mutations were 3 times less frequent in diffuse anaplastic tumors and there were no *MAX* mutations [27]. Even if this skewing did not reach statistical significance, it is likely that *MYCN*/*MAX* mutations bear prognostic value independent of histologically defined risk from diffuse anaplasia.

We had previously shown that—like copy number gains—high *MYCN* expression is correlated with relapse and fatal outcome in a cohort of 102 WT [26]. This could be corroborated in the present study in a larger series of 299 tumors, which further strengthens the possible role of *MYCN* expression as a biomarker in WT stratification. It is conceivable that all three alterations detected for *MYCN*, P44L point mutations, copy number gain, or elevated mRNA expression—which together affect a greater share of WTs—may act in a similar manner and independently contribute to a higher risk of relapse and poor outcome. This should become testable in upcoming larger biobanking and analysis efforts that are under way [28]. Novel methods of culturing Wilms tumors under more physiologic 3D conditions as spheroids or organoids may greatly facilitate such functional studies in future [29, 30].

### Functional role of *MYCN* P44L and *MAX* R60Q

The *MAX* R60Q mutation was proposed to alter DNA binding strength and perhaps dimerization due to its location in the helix-loop-helix domain [2, 27]. It has been found in several other tumor types [8] and in vitro binding assays have indeed confirmed a strongly reduced affinity of the mutant protein for cognate E-box binding sites [31]. In PC12 cells a related R60W mutation and several other *MAX* mutants were shown to have reduced regulatory capacity, observed as repression in that system [32]. This fits to our observation of a reduced transcriptional activation potential of MAX-R60Q compared to the wild-type protein. This likely disturbs the balance of other N-MYC containing transcriptional complexes of the MYC/MAX/MXD1 network. Surprisingly, expression of wild-type or mutant MAX protein in HEK293 cells did not change proliferation in our hands, but the effects may be more subtle or cell type dependent. In line with this, such *MAX* mutations have been described as oncogenic drivers in multiple myeloma, but the mutant tumors showed lower MYC levels and a better prognosis [31].

The *MYCN* P44L mutation remains enigmatic in its functional effects. The mutation is located N-terminal to the conserved Myc-box I, an area that is not represented in 3D structures of N-MYC proteins. In silico prediction by Netphos 2.0 [25] highlighted a potential loss of phosphorylation sites S42 and especially T43 in the mutant protein. Indeed, our detailed MS analyses of tryptic fragments revealed strong phosphorylation at one of the sites, which could not be distinguished based on peptide masses, in the wild-type protein. The mutant protein completely lacked phosphorylation at these positions, while other sites, e.g. the well-known T58 and S62, were phosphorylated equally efficient. Thus, the T58/S62 phosphodegron appears not to be affected. Analyses of protein stability and cell tolerance to overexpression did not reveal substantial differences between both N-MYC versions, accordingly. The lack of conservation of S42/T43 in other MYC paralogues rather calls for a N-MYC specific role of this phosphorylation site and not a general mechanism for all MYC proteins.

### N-MYC interactors

The highly stereotypic proline to leucine mutation together with the concomitant difference in phosphorylation hinted at possibly different binding partners for N-MYC-P44L. Comparative MS analyses of N-MYC containing complexes revealed very similar sets of proteins that were co-purified. When we analyzed the top candidates for differential binding, none of them could unequivocally be reproduced as binding more poorly or better to one of the N-MYC proteins. Thus, at the resolution used in this study, no candidate appears capable to differentially bind wild-type or mutant N-MYC.

The shared interactors detected in our study nevertheless further broaden the set of N-MYC interaction partners in general. While there was clear overlap with known binding partners [19], we expect to have identified in the range of 40–50 further candidates, some of which were already validated by co-IP experiments. The general characteristics of these proteins, i.e. their predicted cellular localization, and their presumed or known biological function make them attractive further candidates to mediate N-MYC effects e.g. in tumor cells.

Dysregulation of several of our candidates (*BMP2K*, *CBLL1*, *DAB2*, *MCRS1*, *FOXK1*, *PEG10* and *YEATS2*) has previously been reported to contribute to different types of cancer [33–39]. Furthermore, their expression levels were correlated with *MYCN* in two cohorts of WT patients and a neuroblastoma data set. For *PEG10* and *YEATS2*, we could validate these correlations using qRT-PCR on a larger cohort. These genes may thus represent additional candidates for prognostic biomarkers or targets in WT.

There are prior reports on the interaction of MYC with FOX proteins and YEATS2. FOXK1, like MYC, regulates several biological processes related to cancer initiation, development, metastasis, angiogenesis, and drug resistance [39, 40], explaining the importance of deregulated FOXK1 in various types of cancers. L-MYC has been reported to interact with FOXK1, and C-MYC has been identified as a FOXK2 and FOXR2 interactor [22, 41]. Myc-boxes II and III appear required to form a ternary FOXR2-MYC/MAX complex and FOXK1 was reported to also bind MAX [42], making a ternary N-MYC/MAX/FOXK1 complex rather likely. Here, we provide evidence for FOXK1 binding to N-MYC via its FHA domain that would join both oncogenic factors.

YEATS2 is a scaffolding subunit of the ATAC complex that is involved in transcriptional activation via its histone acetyltransferase activity [43]. Other components of this complex, like GCN5, have already been reported to bind MYC proteins [44] and a direct interaction of YEATS2 with C-MYC has recently been identified in a high throughput screen [41]. We now show a similar association of YEATS2 with N-MYC. This may contribute to oncogenesis since knockdown of YEATS2 in lung cancer cells resulted in growth suppression, reduced survival, and downregulation of ribosomal protein genes [38], all being key MYC functions.

## Conclusions

We provide further evidence for the negative impact of *MYCN* and *MAX* mutations and elevated *MYCN* expression in WT. While mutant MAX seems to exhibit reduced transcriptional activity, the *MYCN* P44L mutation changes the phosphorylation pattern at the N-terminus with as yet unclear consequences. Future work may address possible differences in protein or chromatin binding of N-MYC proteins carrying phosphomimetic or non-phosphorylatable amino acids. The effect on protein interaction partners seems to be rather subtle. Nevertheless, our analysis of N-MYC containing protein complexes broadens our view on transcriptional regulatory pathways in *MYCN*-driven tumors and provides interesting new biomarker candidates that may be used, perhaps in conjunction with *MYCN*, to improve WT stratification and target selection.

## Supplementary Information

**Additional file 1: Figure S1.** Schematic representation of the human N-MYC and MAX proteins. For N-MYC, the corresponding MYC-boxes (MB) are highlighted. Other functional elements are indicated on the top: transactivation domain (TAD), D element, PEST sequence and nuclear localization signal (NLS). The basic helix-loop-helix (bHLH) and leucine zipper (LZ) domains are involved in dimerization and DNA-binding. Major sites of phosphorylation are identified in blue indicating the amino acid position. The red mark indicates the position of the *MYCN* P44L and *MAX* R60Q mutations.

**Additional file 2: Figure S2.** (A) Growth curves (MTT assay) of HEK293 clones expressing wild-type or mutant HA-N-MYC. The results represent the mean values obtained from biological triplicates. (B) Western blot analysis of HA-N-MYC-expressing HEK293 clones used in (A), showing their endogenous C-MYC and Dox-induced N-MYC expression at day 4 (α-c-Myc/N-Myc (D3N8F) antibody).

**Additional file 3: Figure S3.** IP elutions containing native HA-N-MYC complexes from stably transfected HEK293 pSB-ETiE-HA-MYCN-WT or -P44L cells and untransfected HEK293 control cells (Ctrl), separated by SDS-PAGE and visualized by silver staining. Corresponding immunoblots (below) confirm comparable expression of wild-type and P44L mutant N-MYC.

**Additional file 4: Figure S4.** (A) Immunoprecipitates of HA-N-MYC from HEK293 pSB-ETiE-HA-MYCN-WT or -P44L (WT and P44L) lysates separated by SDS-PAGE and visualized by silver staining. The corresponding immunoblots confirm equal expression of wild-type and P44L N-MYC, as well as MAX, the obligatory N-MYC dimerization partner. (B-C) Extracted ion chromatograms from the phospho-assay, showing the abundance of detected peptides corresponding to the residues F37-W50 of the wild-type (WT) and P44L mutant N-MYC, either unphosphorylated or phosphorylated. The y-axis represents the relative abundance, and the x-axis indicates the elution time. The double peak in the phosphorylated N-MYC-P44L is due to an interruption of the electrospray, leading to a small gap with no signal during the MS.

**Additional file 5: Figure S5.** Expression vectors for doxycycline-dependent expression of wild-type or mutant HA-N-MYC and FLAG-MAX.

**Additional file 6: Table S1**. Summary of mutation screening results and clinical data. **Table S2**. Overlap of WT patients with the *MYCN* P44L and *DROSHA* E1147K mutations. p = p-value (two-tailed Fisher's exact test). **Table S3**. Proteins identified in N-MYC protein complexes. **Table S4**. Cellular component clustering for N-MYC interacting proteins identified in MS. **Table S5**. Statistics of *MYCN*/*YEATS2*/*PEG10* expression in Wilms tumor. **Table S6**. Oligonucleotides for *MYCN* and *MAX* mutation screening. **Table S7**. Oligonucleotides for gene cloning. **Table S8**. Doxycycline concentrations for *MYCN*/*MAX* induction. **Table S9**. Oligonucleotides for real-time PCR. **Table S10**. Antibodies for Western Blot.

## Data Availability

The mass spectrometry proteomics data have been deposited to the ProteomeXchange Consortium via the PRIDE partner repository with the dataset identifier PXD025996.
