# Supplementary figures and images for: MYCN and MAX alterations in Wilms tumor and identification of novel N-MYC interaction partners as biomarker candidates
Source: Cancer Cell Int. 2021 Oct 24;21:555. doi: 10.1186/s12935-021-02259-2 (PMC8543820; doi:10.1186/s12935-021-02259-2)

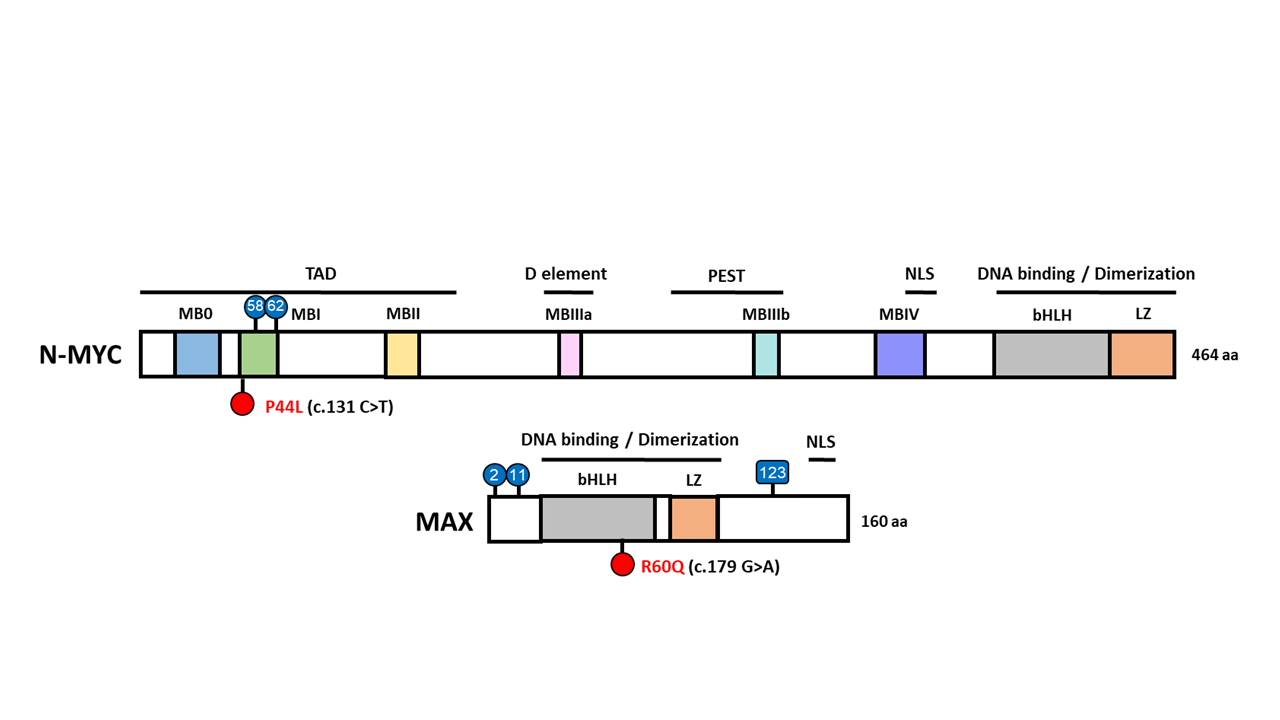

Supplement: Supplementary file 1 — Additional file 1: Figure S1. Schematic representation of the human N-MYC and MAX proteins. For N-MYC, the corresponding MYC-boxes (MB) are highlighted. Other functional elements are indicated on the top: transactivation domain (TAD), D element, PEST sequence and nuclear localization signal (NLS). The basic helix-loop-helix (bHLH) and leucine zipper (LZ) domains are involved in dimerization and DNA-binding. Major sites of phosphorylation are identified in blue indicating the amino acid position. The red mark indicates the position of the MYCN P44L and MAX R60Q mutations. [file 12935_2021_2259_MOESM1_ESM.tif]

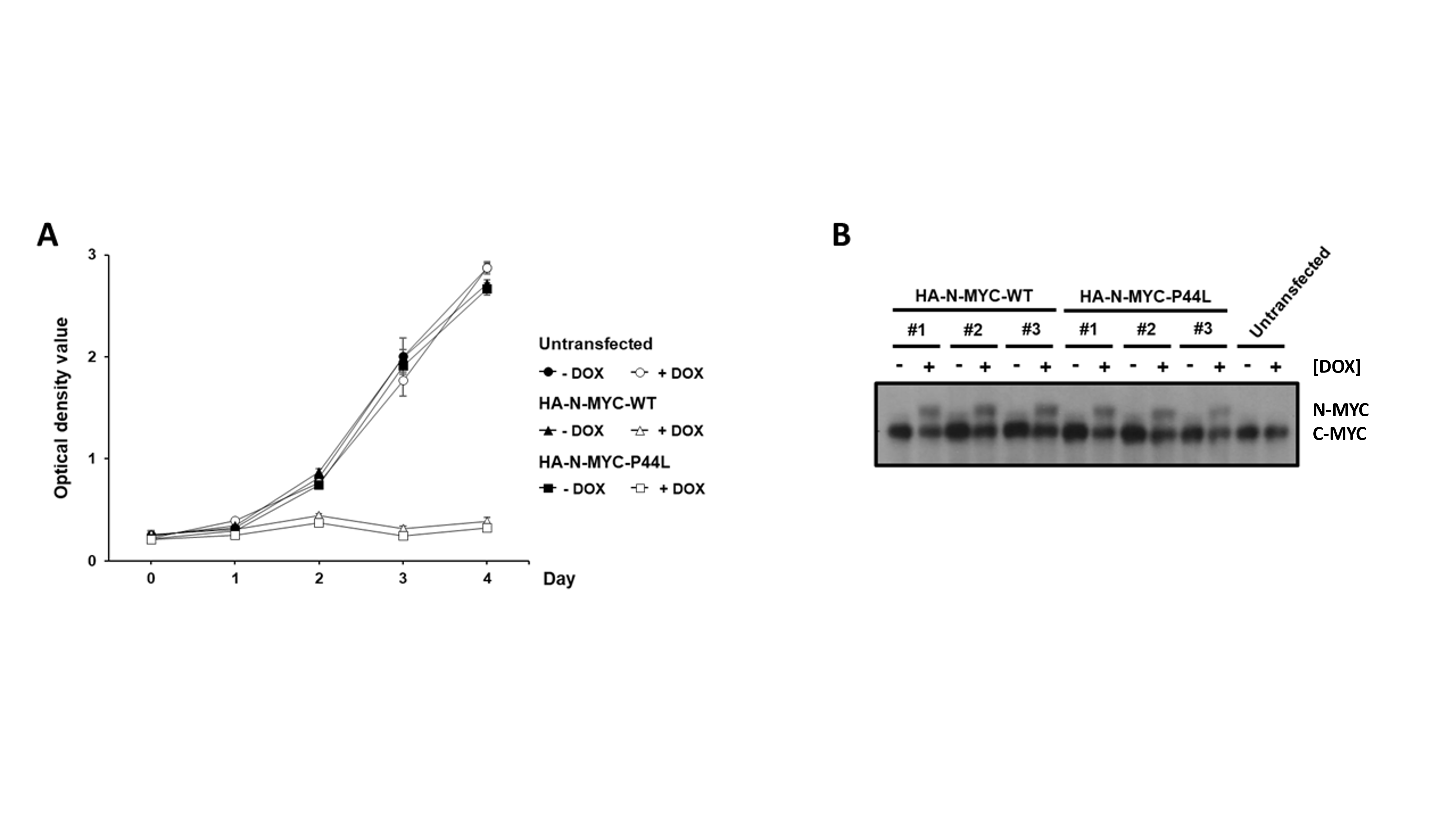

Supplement: Supplementary file 2 — Additional file 2: Figure S2. (A) Growth curves (MTT assay) of HEK293 clones expressing wild-type or mutant HA-N-MYC. The results represent the mean values obtained from biological triplicates. (B) Western blot analysis of HA-N-MYC-expressing HEK293 clones used in (A), showing their endogenous C-MYC and Dox-induced N-MYC expression at day 4 (α-c-Myc/N-Myc (D3N8F) antibody). [file 12935_2021_2259_MOESM2_ESM.tif]

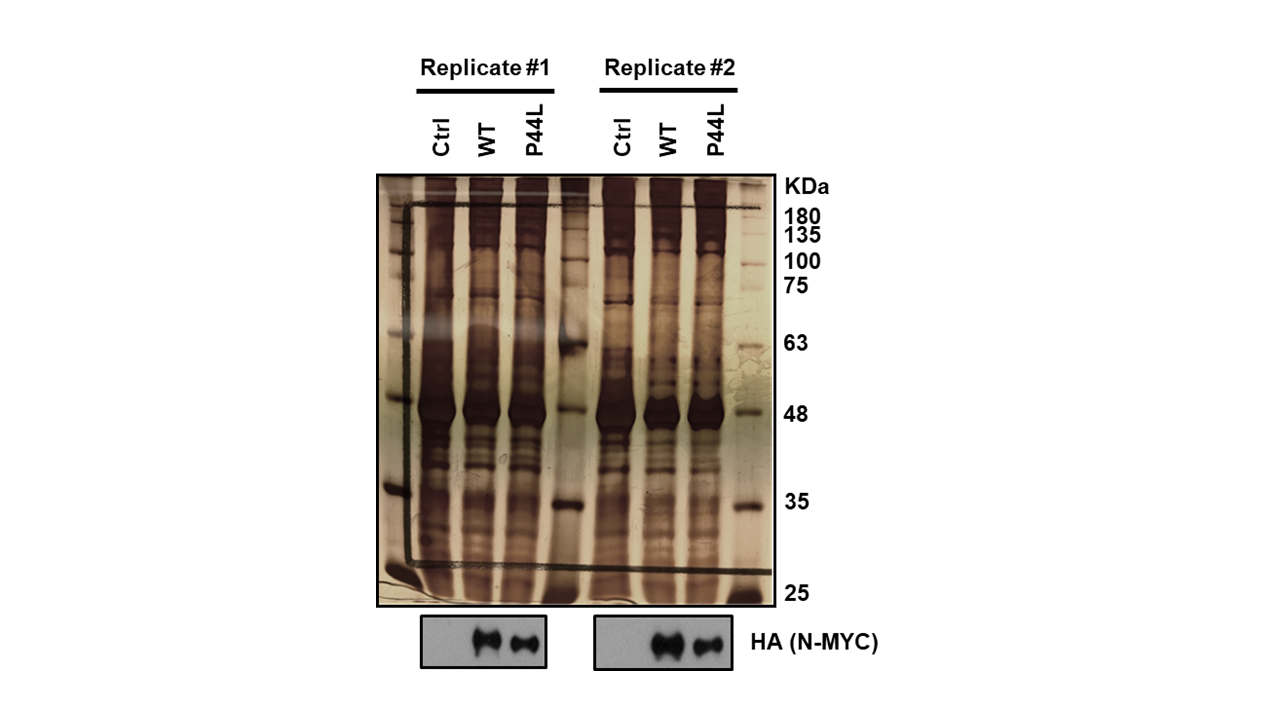

Supplement: Supplementary file 3 — Additional file 3: Figure S3. IP elutions containing native HA-N-MYC complexes from stably transfected HEK293 pSB-ETiE-HA-MYCN-WT or -P44L cells and untransfected HEK293 control cells (Ctrl), separated by SDS-PAGE and visualized by silver staining. Corresponding immunoblots (below) confirm comparable expression of wild-type and P44L mutant N-MYC. [file 12935_2021_2259_MOESM3_ESM.tif]

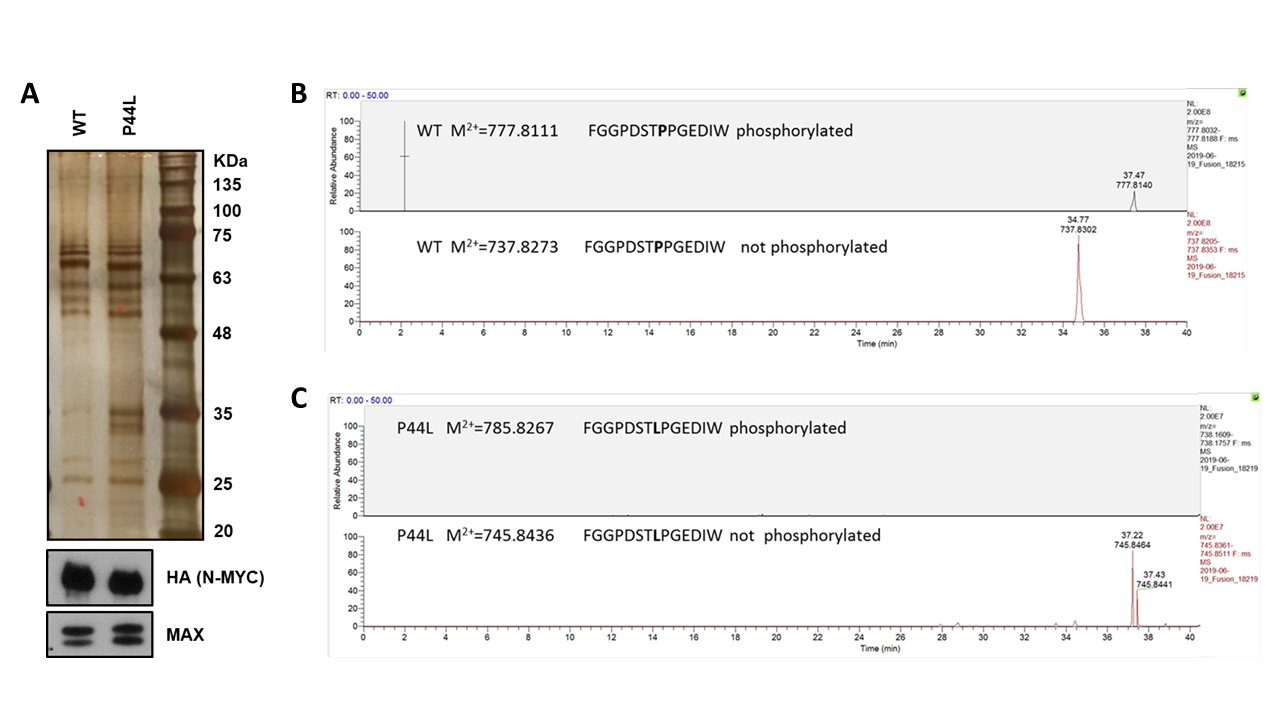

Supplement: Supplementary file 4 — Additional file 4: Figure S4. (A) Immunoprecipitates of HA-N-MYC from HEK293 pSB-ETiE-HA-MYCN-WT or -P44L (WT and P44L) lysates separated by SDS-PAGE and visualized by silver staining. The corresponding immunoblots confirm equal expression of wild-type and P44L N-MYC, as well as MAX, the obligatory N-MYC dimerization partner. (B-C) Extracted ion chromatograms from the phospho-assay, showing the abundance of detected peptides corresponding to the residues F37-W50 of the wild-type (WT) and P44L mutant N-MYC, either unphosphorylated or phosphorylated. The y-axis represents the relative abundance, and the x-axis indicates the elution time. The double peak in the phosphorylated N-MYC-P44L is due to an interruption of the electrospray, leading to a small gap with no signal during the MS. [file 12935_2021_2259_MOESM4_ESM.tif]

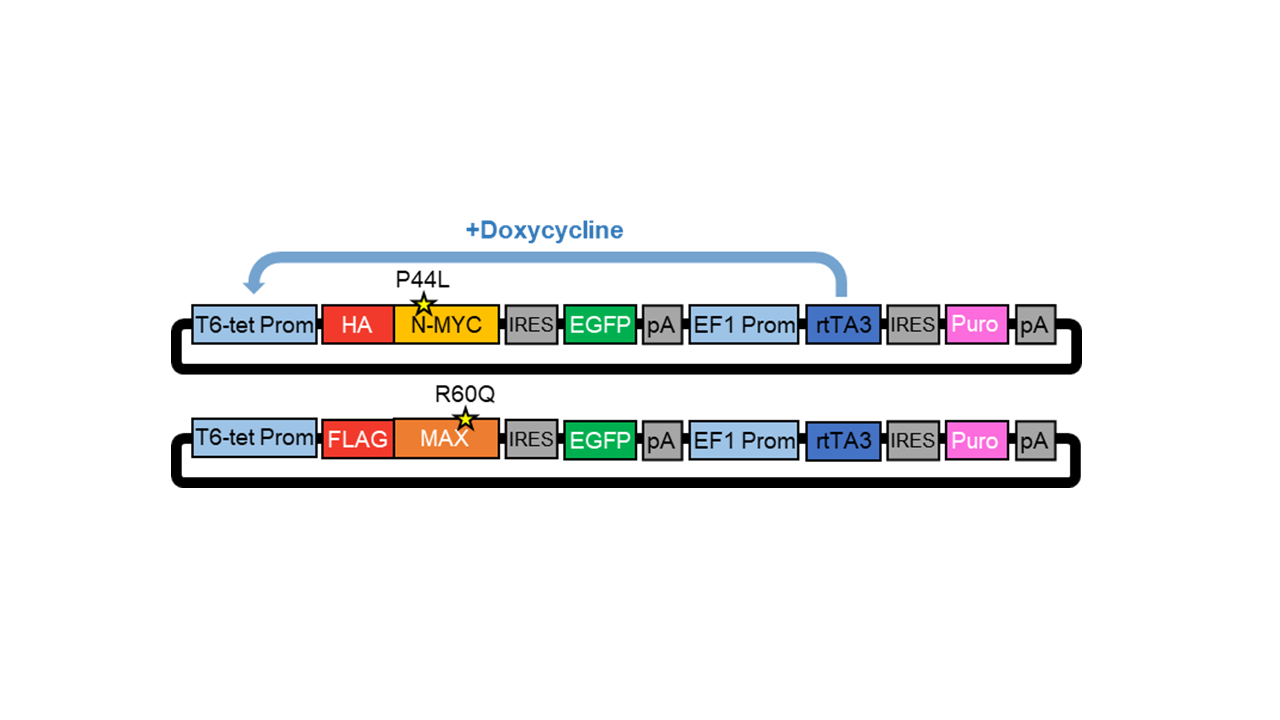

Supplement: Supplementary file 5 — Additional file 5: Figure S5. Expression vectors for doxycycline-dependent expression of wild-type or mutant HA-N-MYC and FLAG-MAX. [file 12935_2021_2259_MOESM5_ESM.tif]
